# Amelioration of Collagen-Induced Arthritis in Female Dark Agouti Rats by Glucosamine Treatment

**DOI:** 10.1155/2013/562905

**Published:** 2013-02-14

**Authors:** Nagaraja Haleagrahara, Dulanthi Tudawe, Srikumar Chakravarthi, Ammu Kutty Radhakrishnan

**Affiliations:** ^1^School of Veterinary and Biomedical Sciences, James Cook University, Townsville, QLD 4811, Australia; ^2^Department of Pathology, International Medical University, 57000 Kuala Lumpur, Malaysia

## Abstract

The present study assessed the therapeutic efficacy of glucosamine hydrochloride against collagen-induced arthritis in female Dark Agouti rats (DA). Arthritis was induced by intradermaly injecting a collagen and complete Freund's adjuvant suspension at multiple sites in the rat at a dose of 4 mg/kg of body weight and thereafter followed by two more boosters of the same dose, after the 1st week and 2nd week of primary immunization. After 21 days from the day of primary immunization, the arthritic group rats were given oral supplementation of glucosamine hydrochloride at a dose of 300 mg/kg of body weight until day 45. The arthritic group treated with glucosamine hydrochloride from day 21 to day 45 showed significant reduction in arthritic histopathological changes of the joints, reduction in paw thickness and also a significant decrease in C-reactive protein and TNF-alpha in the serum. Treatment with 300 mg/kg of glucosamine hydrochloride was able to reverse the arthritic changes, hence suggesting that glucosamine has a therapeutic effect against collagen-induced arthritis.

## 1. Introduction

Rheumatoid arthritis (RA) is a chronic inflammatory autoimmune disease. It is a destructive polyarthropathy that leads to disability and immobility [1]. The main characteristic feature of RA is swelling of the joints, and it is also sometimes manifested in other organ systems in severe disease states. Some of the common physical symptoms of RA are swelling of joint areas, painful and stiff joints, fatigue, malaise, weight, loss and sometimes even depression [2]. The exact aetiopathogenesis of RA is not known. Risk factors such as genetic predisposition and environmental factors like cigarette smoking [3], microbial infections [4], and blood transfusions [5] will lead to the activation of CD 4^+^ T helper cells, which will lead to the stimulation of various cells in the joints that produce cytokines and inflammatory mediators that play an important role in the development of RA. Inflammatory mediators like matrix metalloproteinases (MMP) and cell adhesion molecules (CAMs), together with cytokines like tumor necrosis factor-alpha (TNF-*α*) and interleukin-1 (IL-1), lead to joint destruction in RA patients [2, 6].

At present, there are few treatment options available for RA. Nonsteroidal anti-inflammatory drugs (NSAIDs), cyclooxygenase-2-selective NSAIDs [7], glucocorticoids, biological response modifying agents, and disease modifying antirheumatic drugs (DMARDs) are prescribed by physicians currently as single therapy or combined multiple drug therapy [8]. It has beenshown to slow the disease progression in some cases, but itis not shown to completely reverse symptoms of RA and prevent the disease onset.

Glucosamine is an amino monosaccharide, which is a derivative of glucose. It is naturally found in mucous secretions, connective tissue, skin, tendons, and ligaments, and it is found in highest concentration in cartilage [9]. With age, the production of glucosamine by the body will decrease; hence, it needs to be replenished by the use of oral glucosamine supplements. Currently glucosamine is used in the therapy for osteoarthritis (OA) to synthesize glycosaminoglycans (GAGs) [10]. Studies have also shown that glucosamine supplementation can repair the cartilage to some extent in OA [11]. Recent studies have also shown that glucosamine has immunosuppressive properties, which can successfully suppress the activity of T-lymphocytes and dendritic cells [12] which are involved in adaptive immune responses. The present study investigated the efficacy of glucosamine against collagen-induced arthritis in female Dark Agouti (DA) rats.

## 2. Methods

### 2.1. Reagents

Glucosamine hydrochloride (D-(+)-glucosamine hydrochloride; G4875; C_6_H_13_NO_5_ *·*HCl; mol wt:215.63 g/mol; Sigma), complete Freund's adjuvant (CFA), type II collagen, and all other analytical chemicals used for the experiment were obtained from Sigma Aldrich (St. Louis, MO, USA). The dose received by the treatment group in this study was 300 mg/kg/rat [13].

### 2.2. Animals

Female Dark Agouti (DA) rats [14] of 6–10 weeks old were obtained from the Institute of Medical Research, Kuala Lumpur, Malaysia. The rats were kept in individual ventilation cages throughout the study. Rats were housed in standard laboratory conditions with 12:12 light-dark cycle with free access to food and water throughout the study. There were three groups of animals in this study, each group containing 10 rats. Group 1—control without any treatment, Group 2—arthritis only group, and Group 3—arthritis with glucosamine hydrochloride (300 mg/kg/rat). All the experimental procedures were in accordance with the guidelines for the care and use of laboratory animals, and approval from the Institutional Research and Ethics Committee was taken prior to the experiments.

### 2.3. Collagen-Induced Arthritis

Type II collagen from chicken tracheal cartilage obtained from Sigma Aldrich (USA) was dissolved in 0.1 M of cold acetic acid. The dissolved collagen was then emulsified with an equal volume of complete Freund's adjuvant (CFA) (Sigma Aldrich, USA). Each rat was injected intradermally with 4 mg/kg of collagen suspension at multiple joint areas [15] and also at the base of the tail [16]. A booster dose injection was given after a week following primary immunization, and a second booster was administered 15 days after first booster injection.

### 2.4. Treatment of Animals with Glucosamine Hydrochloride

Glucosamine hydrochloride was administered orally using a rodent feeding tube with a dose of 300 mg/kg/rat. The treatment was started 21 days after the primary immunization and continued until day 45.

### 2.5. Measurement of Body Weight and Paw Thickness Changes

The severity of arthritis in the animals was evaluated by quantifying the changes in paw thickness in the front and hind paws. Thickness was measured using a digital caliper once every five days (there was not any significant change in the paw thickness during the 5-day period in the earlier studies done in our laboratory) until day 45. The body weight changes of the rats were also recorded once every five days during the study period.

### 2.6. Collection of Plasma

At the end of the experimental period, animals were anesthetized with sodium pentobarbital (40 mg/kg body weight) and blood samples were collected by cardiac puncture. A volume of 3-4 mL of blood was collected in a vacutainer tube which contained EDTA. These tubes were then centrifuged at 5000 rpm for 10 minutes at 4°C. The plasma was separated and stored at −30°C for further investigations. Rats were sacrificed with an overdose of anesthesia, and limbs were harvested for joint histopathology.

### 2.7. C-Reactive Protein and Tumour Necrosis Factor-Alpha Assay

The C-reactive protein (CRP) and tumour necrosis factor-alpha (TNF-*α*) concentration level in the plasma were quantified using a commercial kit according to the standard protocol recommended by the manufacturer (Assay Pro, USA, and eBioscience, USA). In the C-reactive protein assay, 25 *μ*L of the standards was added to the relevant wells, and 25 *μ*L of the diluted samples were added in duplicates. Biotinylated rat CRP (25 *μ*L) was added to all wells, gently mixed, and kept for incubation for 2 h. After incubation, the plate was washed using wash buffer and 50 *μ*L of diluted streptavidin peroxidase conjugate was added and incubated for 30 min. The plate was washed and filled with 50 *μ*L chromogen substrate and left for 10 min for color development. A stop solution was added, and the absorbance produced was immediately read at 450 nm. The CRP concentration of each of the samples was calculated in *μ*g/mL based on the standard curve obtained. The concentration of TNF-*α* in the plasma was quantified using a commercial rat TNF-*α* ELISA kit. The previous night, the plate was coated with diluted capture antibody as described in the study protocol provided with this kit. Then, 100 *μ*L of the standards were added to the standard wells and 100 *μ*L of samples was added to the sample's wells in duplicates. The plate was incubated at room temperature for 2 h. After washing with buffer, 100 *μ*L of the diluted detection antibody solution was added to all wells, and the plate was incubated for 1 h at room temperature. Afterwards, 100 *μ*L of the diluted avidin HRP was added to all the wells and incubated at room temperature for 30 min. Following this, 100 *μ*L of the substrate solution was added to all the wells. Finally, 50 *μ*L of 2NH_2_SO_4_ was added and the absorbance was read at 450 nm.

### 2.8. Histopathological Analysis

The flesh from the joints was cleared away, and the joint sample was stored in 10% formalin solution for three weeks in specimen bottles. The joint samples were stored in a decalcification agent for 48 hours before processing. The decalcified samples were processed using Leica TP 1020 tissue processor machine and embedded in paraffin blocks using a Leica EG 1160 tissue embedding center. The haematoxylin and eosin stained slides were visualized and analyzed using a Nikon Eclipse 80i microscope. The joints were evaluated and analyzed according to a grading system adopted from a previous study [17] (Table 1).

### 2.9. Statistical Analysis

Statistical analysis was carried out by one-way analysis of variance (ANOVA) using the SPSS. The histology of the joints and the plasma levels of C-reactive protein and TNF-*μ* were also statistically analyzed for nonparametric measures using the Mann-Whitney *U* test. *P* < 0.05 was considered statistically significant.

## 3. Results

### 3.1. Effects on the Body Weight

**Figure 1:**
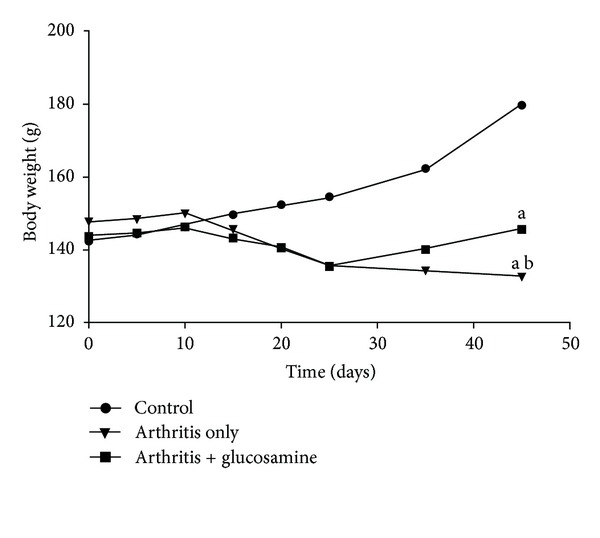
Changes in body weight in the two experimental groups. (a) *P* < 0.05—control with other groups; (b) *P* < 0.05—arthritis with arthritis + glucosamine.

In the arthritis only group, there was a significant decrease in the body weight (*P* < 0.05). Arthritis rats that had oral supplementation of glucosamine showed a steady increase in the body weight initially, but there was a significant decrease in the weight gain after 15 days until day 25, but weight increased significantly after day 25 in these rats. Even though there was an increase in the body weight with glucosamine treatment, the weight change did not increase significantly compared to control rats (Figure 1).

### 3.2. Effects on Gross Arthritic Symptoms

**Figure 2:**
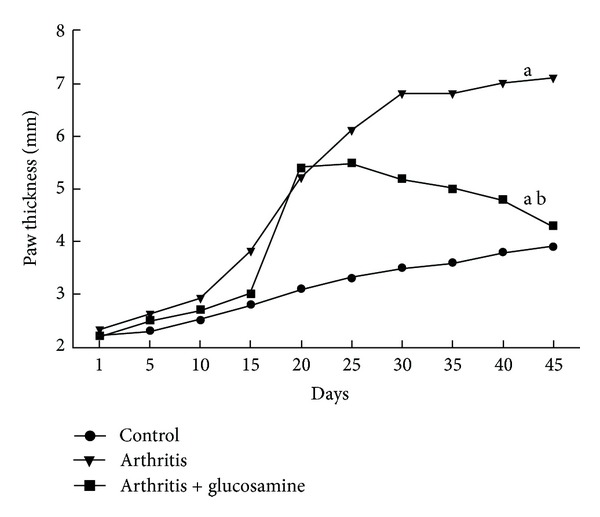
Changes in paw thickness. (a) *P* < 0.05—control with other groups; (b) *P* < 0.05—arthritis with arthritis + glucosamine.

**Figure 3:**
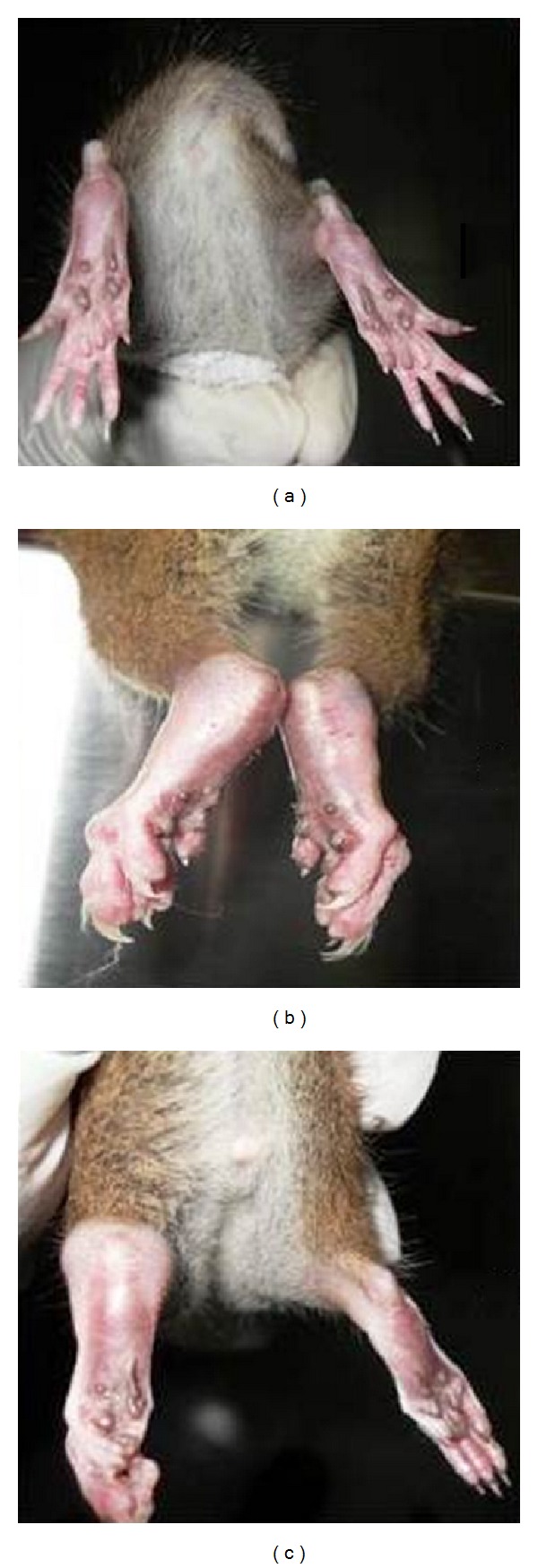
Paw edema—(a) hind paw and ankle of control rats; (b) arthritis control showing severe swelling at the joints in both ankles; (c) hind paw and ankle joint of arthritic rat with glucosamine.

A significant increase (*P* < 0.05) in the hind paw thickness was observed in the arthritic rats from day 1 to day 45 when compared to control rats. Glucosamine treatment depicted a significant reduction (*P* < 0.05) in the paw thickness compared to arthritis alone at the 45th day. There was severe swelling of the joints in the hind paw in the arthritic rats. The joints showed redness and ankylosis. The group of rats that were started on oral supplementation of glucosamine showed only mild to moderate signs and symptoms of arthritis, when compared to the arthritis alone group. The swelling of the ankle joints in the hind paw was significantly less in the treated group than in the arthritis only group (Figures 2 and 3).

### 3.3. Effects of Glucosamine on C-Reactive Protein

**Figure 4:**
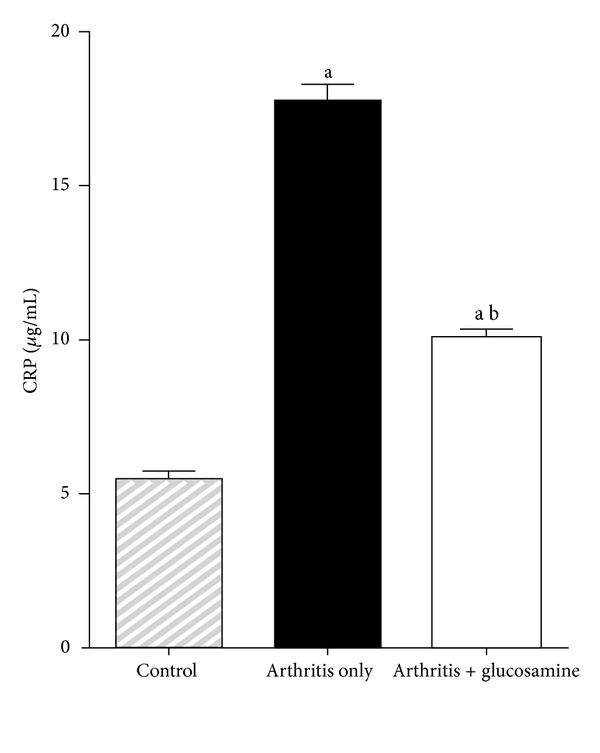
Plasma concentration of C-reactive protein. (a) *P* < 0.05—control with other groups; (b) *P* < 0.05—arthritis with arthritis + glucosamine.

A significant increase (*P* < 0.05) in C-reactive protein level was seen in arthritis only rats until 45 days compared to control rats. The level of C-reactive protein concentration was significantly reduced (*P* < 0.05) after oral supplementation of glucosamine (Figure 4).

### 3.4. Effects of Glucosamine on TNF-*α*

**Figure 5:**
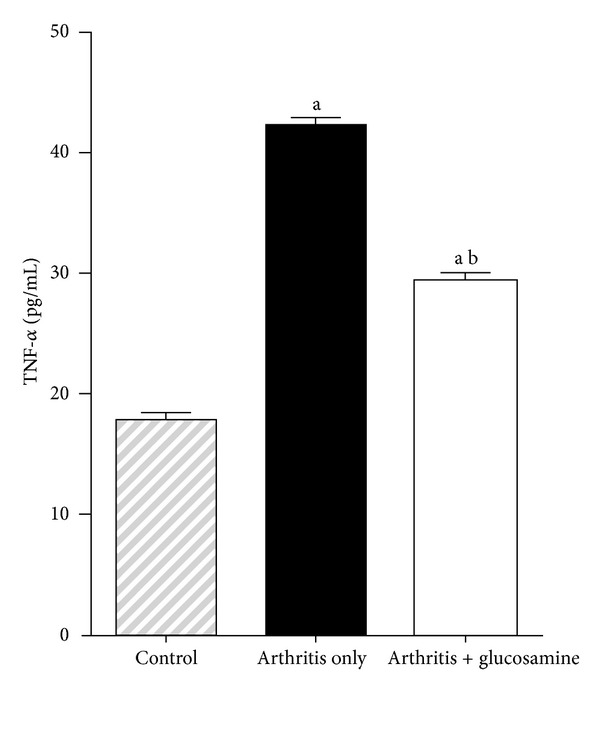
Plasma levels of TNF-*α*. (a) *P* < 0.05—control with other groups; (b) *P* < 0.05—arthritis with arthritis + glucosamine.

A significant increase (*P* < 0.05) in the TNF-*α* level was seen in the arthritis alone rats compared to controls. There was a significant decrease in TNF-*α* levels in the plasma of the glucosamine treated group (*P* < 0.05) than the untreated arthritis only control group. Glucosamine has reduced the concentration of this inflammatory marker significantly in the treated group (Figure 5).

### 3.5. Histopathological Changes

To further evaluate the treatment aspect of glucosamine hydrochloride against rheumatoid arthritis, histopathological analysis was done as described in Table 1, which is a method adapted from the previous study [17]. Arthritis only group showed a severity of grade three, while the glucosamine treated arthritic group had pathological characteristics of grade 1–0 severities (Figure 5).

**Figure 6:**
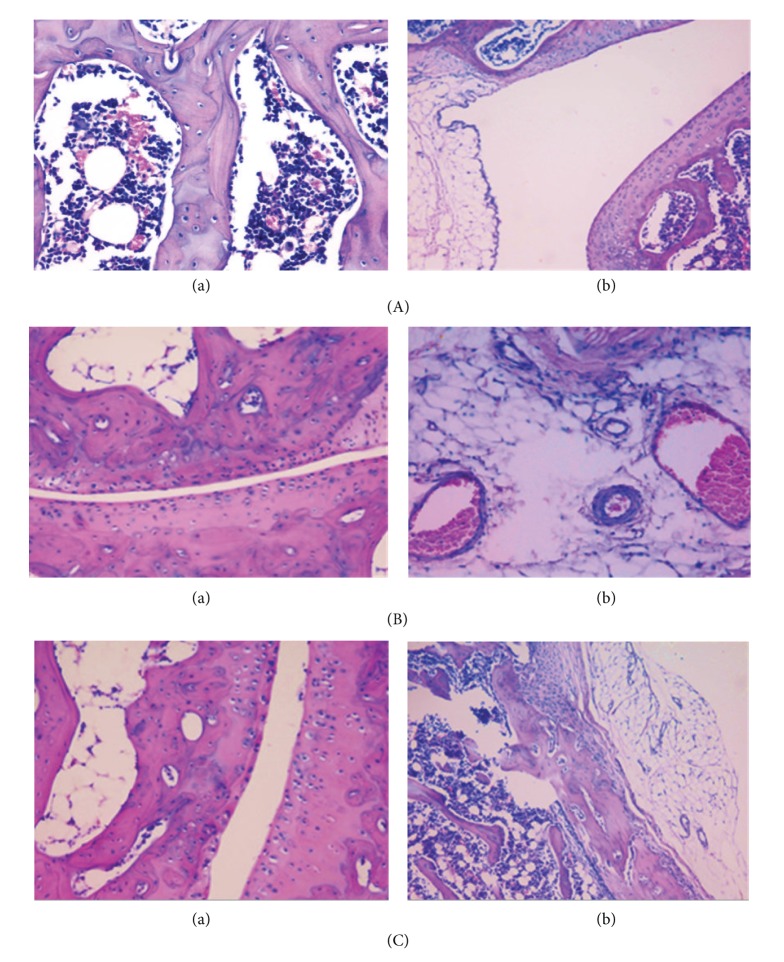
Histopathological analysis of joint morphology (200x). Control synovium (Aa) and joint (Ab); arthritis joint narrowing (Ba) and synovial congestion (Bb); arthritis with glucosamine joint (Ca) and reduced synovial congestion (Cb).

Synovial hyperplasia is observed in the untreated arthritis only group, which appeared to have developed extensive oedema causing the joint space to narrow. The group treated with glucosamine showed the best results, that is, the reversal of synovial hyperplasia as in these rats and the joint appeared to be similar to a normal joint (Figure 6). There were healthy regenerative synovium and areas of fibrosis, angiogenesis, and fibroblasts proliferation with accumulation of collagen, suggesting good wound healing. In the untreated arthritis rats, the joints had extensive oedema with narrowing of the space. The surface of the joint margins showed degenerative changes. In the glucosamine treated arthritic groups, the synovial space was adequate with focal areas of degenerative changes (Figure 6).

In the untreated arthritic group, the synovium showed hyperplasia and increased angiogenesis and fibrosis. The joint vascularity showed congestion with dilated blood vessels. The group treated with glucosamine showed no synovial congestion compared to the other two groups (Figure 6). There was an extensive inflammation with pannus formation in several places among untreated arthritic rats. The pannus was composed of a granulomatous accumulation of chronic inflammatory cells like lymphocytes, plasma cells, and macrophages and multinucleate giant cells seen in the untreated arthritic group. The glucosamine treated group had mild to moderate inflammation (Figure 6).

## 4. Discussion

Collage-induced arthritis (CIA) was induced when type II collagen was emulsified with complete Freund's adjuvant (CFA) and injected into the base of the rat's tail. In this animal model of arthritis study, female Dark Agouti or DA rats, aged 6–10 weeks, are used. Complete Freund's adjuvant injection will induce an immunological hypersensitivity reaction to collagen in the rats, leading to the development of systemic effects of chronic rheumatoid arthritis [18]; therefore, the collagen-induced arthritis DA model shows most similarities to the human rheumatoid arthritis. Dark Agouti rats are especially susceptible to rheumatoid arthritis. These rats develop a chronic and severe form of autoimmune arthritis, which leads to permanent bone destruction and ankylosis of affected joints. Arthritis-induced joint destruction in DA rats is believed to be the consequence of cell mediated immune responses, which mimic the pathogenesis of human rheumatoid arthritis [19–21].

This study evaluated the oral administration of glucosamine on histopathological and inflammatory markers. The dose of glucosamine used in this study is 300 mg/kg body weight, which would give an approximate serum level of glucosamine (0.05 mM) similar to that in humans [13, 22]. Because of the substantial amount of gastrointestinal loss in rats, a higher daily dose of glucosamine is usually selected in animal models of arthritis. Glucosamine is a naturally occurring chemical component that is found in its highest concentration in joint areas [23, 24].

Our study showed a significant decrease in body weight around the 25th day in arthritis rats as compared to the normal control. Glucosamine treatment (300 mg/kg) showed significant recovery in the body weight during the last 10 days of the treatment when compared to their arthritic controls. Glucosamine treatment also suppressed the paw thickness from day 20 to day 45. Glucosamine acts at the joints by being incorporated by the chondrocytes into the cartilage. Here it will stimulate the synthesis of physiological proteoglycans and also will decrease the activity of matrix metalloproteinases, which are catabolic enzymes that attack the cartilage. Glucosamine can also inhibit the degradation of cartilage, which is induced through IL-1 [21, 25]. Thus, glucosamine acts as a substrate for the biosynthesis of mucopolysaccharides in bones and joints, which will aid in restoring damaged cartilage.

Several studies have implicated TNF-*α* as a contributor to cellular damage in collagen-induced arthritis. The increased concentration of this cytokine in circulation can be interpreted as a progression of cartilage cell injury [26, 27]. The anti-inflammatory action of glucosamine might have lowered circulating TNF-*α* concentration and consequently reduced the joint damage. C-reactive protein is produced under conditions of inflammation in cartilage and bone and is a useful biomarker in the evaluation of disease progression and response to therapeutic intervention in a number of inflammatory disorders, including RA. Higher concentration of C-reactive protein will point toward increased joint changes in arthritis [28]. In our study, it is shown that glucosamine reversed inflammatory damage in joints when compared with untreated arthritic rats.

Histopathological changes in our study correlated with macroscopic observations, including changes in the paw thickness. Arthritis only rats showed maximum degenerative changes. A significant reduction in the joint changes was observed in glucosamine treated groups. In the treated rats, a reversal of many of the arthritis-induced histological changes was observed. In terms of the joint analysis grading system, the untreated arthritic rats mostly showed the grade 3 characteristics, which are of the most severity level, and the glucosamine treated group showed the least severity (grade 1-2). Observed protective effects may be due to the inhibition of inflammatory mediators by glucosamine [12].

Glucosamine acts at the joints and is known to efficiently suppress the activity of T-lymphocytes and dendritic cells, two crucial cells involved in immune responses [12]. Tumour necrosis factor-*α* (TNF-*α*) and interleukin-1 (IL-1) are produced by macrophages and synovial lining cells and can be found in high concentrations in arthritis. Both of these cytokines act synergistically in the production of matrix metalloproteinase (MMP), expression of various cell adhesion molecules (CAMs), and secretion of prostaglandins [6, 29]. The observed joint destruction could be because of the accumulation of MMPs, and prostaglandins and glucosamine treatment has suppressed the productions of these inflammatory mediators. By suppressing joint inflammatory responses, glucosamine prevents pannus formation. During arthritis, the pannus becomes fibrosed and will have minimal vascularization, and this will result in the destruction of the cartilage. Histopathology revealed reduced pannus and bony ankylosis in DA rats with glucosamine treatment confirming the protective effect of glucosamine against joint damage. This supports some of the other studies that have shown that glucosamine supplementation can repair cartilage damage to some extent in osteoarthritis patients [10, 11].

Thus, our study evaluated the effect of oral administration of glucosamine to Dark Agouti rats on gross, biochemical, and histopathological changes. Glucosamine treatment not only suppressed joint swelling, it also reduced the inflammatory mediators like TNF-*α* and C-reactive proteins. There was reduced histopathological change in the joints. These observations support the view that glucosamine suppresses the chronic inflammatory phase in collagen-induced arthritis. Glucosamine exerts its anti-inflammatory effects by suppressing the activation of inflammatory cytokines. A rapid and significant beneficial effect of glucosamine was observed against arthritis in DA rats in this study. Thus, the findings confirmed the joint modifying and anti-inflammatory properties of glucosamine in collagen-induced arthritis.

## 5. Conclusion

In conclusion, the findings from this study demonstrate that glucosamine effectively reduces the degree of edema and inflammation caused by collagen-induced arthritis in female Dark Agouti rats. Results also confirm glucosamine as a therapeutic agent for the treatment of rheumatoid arthritis. A dose-response study of glucosamine-induced anti-inflammatory effects and its molecular mechanisms are being investigated further.

## Figures and Tables

**Table 1:** The histopathological characteristics observed in a joint affected by rheumatoid arthritis and the grading system for the analysis of joints.

	0	1	2	3
Oedema	Nil	Mild	Moderate	Severe
Cellular Infiltration	Nil	Mild scattered infiltration	Lymphocytes and macrophages	Sheets of inflammatory cells/granulomas/MNG cells
Joint space	No narrowing	Mild narrowing	Moderate narrowing with very little intermeningeal space	Total obliteration of joint space
Synovial hyperplasia	Nil	Mild	Moderate	Extensive
Fibrosis	Nil	Mild	Moderate	Extensive/severe
Erosion	Absent	Present		
